# Adenosine Receptor Modulates Permissiveness of Baculovirus (Budded Virus) Infection via Regulation of Energy Metabolism in *Bombyx mori*

**DOI:** 10.3389/fimmu.2020.00763

**Published:** 2020-04-28

**Authors:** Yu-Hsien Lin, Chia-Chi Tai, Václav Brož, Cheng-Kang Tang, Ping Chen, Carol P. Wu, Cheng-Hsun Li, Yueh-Lung Wu

**Affiliations:** ^1^Biology Centre of the Czech Academy of Science, Institute of Entomology, Ceske Budejovice, Czechia; ^2^Faculty of Science, University of South Bohemia, Ceske Budejovice, Czechia; ^3^Department of Entomology, National Taiwan University, Taipei, Taiwan

**Keywords:** glycolysis, baculovirus, adenosine s*i*gnaling, gloverin, *Bombyx mori*, *Spodoptera frugiperda*

## Abstract

Although the modulation of host physiology has been interpreted as an essential process supporting baculovirus propagation, the requirement of energy supply for host antivirus reactions could not be ruled out. Our present study showed that metabolic induction upon AcMNPV (budded virus) infection of *Bombyx mori* stimulated virus clearance and production of the antivirus protein, gloverin. In addition, we demonstrated that adenosine receptor signaling (AdoR) played an important role in regulating such metabolic reprogramming upon baculovirus infection. By using a second lepidopteran model, *Spodoptera frugiperda* Sf-21 cells, we demonstrated that the glycolytic induction regulated by adenosine signaling was a conservative mechanism modulating the permissiveness of baculovirus infection. Another interesting finding in our present study is that both BmNPV and AcMNPV infection cause metabolic activation, but it appears that BmNPV infection moderates the level of ATP production, which is in contrast to a dramatic increase upon AcMNPV infection. We identified potential *AdoR* miRNAs induced by BmNPV infection and concluded that BmNPV may attempt to minimize metabolic activation by suppressing adenosine signaling and further decreasing the host's anti-baculovirus response. Our present study shows that activation of energy synthesis by adenosine signaling upon baculovirus infection is a host physiological response that is essential for supporting the innate immune response against infection.

## Introduction

Baculoviruses are double-stranded circular DNA viruses with genomes of ~80–180 kb. Baculoviruses can infect many species of arthropods, among which lepidopteran larvae are the most common host (1, 2). *Autographa californica* nucleopolyhedrovirus (AcMNPV) is the most thoroughly studied baculovirus, and it has been established as the primary baculovirus expression system since the 1980's (3). Another commonly studied baculovirus is *Bombyx mori* nucleopolyhedrovirus (BmNPV), which is also used to express exogenous recombinant proteins (4). Although AcMNPV and BmNPV have highly similar genetic structures, they have very different host ranges (5, 6). AcMNPV is able to infect the broader range of lepidopteran larvae but has a lower capacity to infect *B*. *mori*, whereas BmNPV can only infect *B. mori* and is not capable of infecting the larvae of other Lepidoptera species (1, 7, 8).

Baculovirus infection has significant impacts on host physiology, establishing optimal conditions for successful propagation. Several virus-encoded proteins or microRNAs that regulate the host cell cycle, apoptosis, cytoskeleton rearrangement, immune responses, and membrane receptors have been reported for different baculoviruses (9). In addition, virus growth relies heavily on host resources, and the distribution and transfer of energy in hosts are important factors that affect viral replication. Studies of BmNPV and AcMNPV have demonstrated that baculovirus infection significantly increases the oxygen consumption and tricarboxylic acid (TCA) cycle activity in the permissive host (10–12). Increased expression of metabolic pathway genes, such as citrate synthase and pyruvate dehydrogenase, as well as genes involved in mitochondrial respiration, has been observed in AcMNPV- and BmNPV-infected cells (13).

Although intensification of host biosynthesis by viruses can provide sufficient substrates for virus replication, the host can also modulate its own metabolic activity to restrict viral propagation. For example, expression of *samhd1* in human myeloid cells decreases the dNTP pool, limiting reverse transcription and suppressing virus replication (14), and induction of interferon upon virus infection disrupts sterol biosynthesis and suppresses viral replication (15). In addition, increased metabolic activity during infection might prompt the immune response against pathogens. Transcriptomic and biochemical studies in fruit fly, tobacco budworm, cockroach, and mosquito demonstrate that genes involved in energy synthesis, detoxification, and carbohydrate metabolism are upregulated upon bacterial or fungal infection and that inhibition of host carbohydrate metabolism decreases the immune response against pathogens (16–20). The molecular mechanism involved in the systematic switch of metabolic homeostasis upon infection was described recently in *Drosophila melanogaster*. Upon bacterial and parasitic wasp infection, *Drosophila* immune cells release adenosine as a signal to activate metabolic reprogramming, which shifts energy distribution from developmental processes toward the immune response (19, 21).

Although previous studies have reported that energy production is induced after infection in both BmNPV-infected BmN cells and AcMNPV-infected Sf-9 cells, it is unclear whether this phenomenon is restricted to permissive infection conditions (11, 12). Moreover, it could not be ruled out that metabolic induction might contribute to the host immune response against virus infection. Therefore, in this study, we compared the metabolic responses of BmN cells and *B. mori* larvae upon non-permissive (by AcMNPV) and permissive (by BmNPV) infection conditions. We also performed functional analysis by inhibiting glycolysis with 2-deoxy-D-glucose (2DG) treatment and examined the baculovirus infective capacity. Furthermore, through reverse genetic and pharmaceutical approaches, we identified that adenosine signaling is a conserved mechanism that regulates metabolic activation and gloverin expressions upon AcMNPV infection.

## Materials and Methods

### *B. mori* Larvae, and Cells

*B. mori* strain is a tetramolted hybrid of (Kou × Fu) × (Nung × Feng) generated by Taiwan Sericultural Improvement Station, Miaoli, Taiwan. Larvae were fed mulberry leaves and housed in a growth chamber at a constant temperature of 26°C with a photoperiod of 16 h of light and 8 h of darkness (22).

The *S. frugiperda* cell line IPLB-Sf-21 and *B. mori* larval ovarian cell line BmN were cultured in TC-100 insect medium containing 10% fetal bovine serum (Gibco BRL) in an incubator at 26°C (1).

### Titration of Budded Virus

Sf-21 and BmN cells were used for the reproduction of recombinant AcMNPV and BmNPV budded virus carrying the enhanced green fluorescent protein gene, respectively, TCID50 (50% tissue culture infectious dose) values and real-time quantitative PCR (RT-qPCR) were used to estimate viral titers (1, 23).

### Nucleic Acid Extraction

RNA from infected cells (2 ×10^5^ cells/well) or larvae was extracted using the TRIzol reagent (Invitrogen). Two third-instar larvae were pooled together for homogenization. OD values and RNA concentrations were detected using a microvolume spectrophotometer (Nanodrop 2000; Thermo Scientific) (24, 25). cDNA was synthesized using the PrimeScript™ RT reagent kit (Takara). Briefly, 500 ng of RNA was dissolved in ddH_2_O (total volume of 6.5 μL), after which 2 μL of 5 × PrimeScript™ buffer, 0.5 μL of RT enzyme mix, 0.5 μL of oligo dT primers and 0.5 μL of random 6-mers (total volume, 10 μL) were added according to the manufacturer's instructions. The mixture was incubated at 37°C for 15 min for reverse transcription, after which the reaction was terminated by heating at 85°C for 5 s. The obtained product was stored at 4°C for subsequent analysis (1). The cDNA was quantified using a Nanodrop 2000 spectrophotometer.

### Analysis of Gene Expression by RT-qPCR

RT-qPCR carried out with SYBR green (Bioline) and the ABI PlusOne real-time system (StepOnePlusTM, Applied Biosystems) was used for relative target gene quantification. Each sample contained 10 μL of SYBR green, 0.8 μL of primer, 1 μL of cDNA, and ddH_2_O to adjust the total volume to 20 μL. A list of primer sequences used in this study is given in Table S1.

### siRNA Cell Transfection

siRNAs were synthesized by MDBio Co. (siRNA-AdoR sequence: 5′-GCGUCU UGUUAGCUGCUUU-3′; siRNA-control sequence: 5′-AAUUCUCCGAACGUGUC ACGU-3′). BmN cells were seeded into a 24-well plate at a density of 2 × 105 cells per well and the cells were transfected with siRNAs (100 pmol) using the Lipofectamine RNAiMAX Reagent (Invitrogen). RT-PCR analysis was carried out to determine the inhibition efficiency of siRNA-transfected cells at 48 h post transfection (hpt). After transfection for 24 h, AcMNPV and BmNPV infections were separately carried out at a multiplicity of infection (MOI) of 1. The cells and supernatants were harvested to detect viral titers and ATP levels at 48 h postinfection (1).

### Pharmacological Treatment of BmN Cells, Sf-21 Cells, and Larvae

BmN and Sf-21 cells (2 × 10^5^) were preincubated for 2 h with Dipy (20 μM), 2DG (10 mM), or adenosine (100 μM). Subsequently, the cells were infected with AcMNPV or BmNPV at a MOI of 1. Third-instar larvae were injected with AcMNPV (1 × 10^6^ PFU/5 μL) and Dipy (20 mM, 5 μL) or AcMNPV (1 × 10^6^ PFU/5 μL) and 2DG (0.5 mM, 5 μL). The supernatants or hemolymphs were harvested to detect viral titers and ATP levels at 48 h postinfection.

To assess the cytotoxicity, we treated the BmN and Sf-21 cells (2 × 10^5^) with DMSO (0.05%), 2DG (10 mM), Dipy (20 μM) and adenosine (10 mM) for 24, 48, and 72 h in 12 well-pates, and cells were stained with propidium iodide (50 μg/mL). for labeling the dead cells. The quantification of live and dead cells was conducted by flow cytometry in the 585 ± 40 nM channel using ACEA NovoCyte™ 3,000, and 10,000 events were quantified for comparison. The results were shown in Figure S1.

### *B. mori* Hemolymph Collection

Late third-instar larvae of *B*. *mori* were first placed in a −20°C freezer for 2 min to prevent the secretion of defensive fluids. The larval prolegs were cut off, and 10 μL hemolymph from one late third-instar larva was collected with a pipette and transferred to 1.5-mL centrifuge tubes. Hemocytes were removed by centrifugation at 3,000 × *g* for 1 min (26), after which the supernatant was collected to measure the ATP level. For the glucose, trehalose, and adenosine measurements, 10 μL hemolymph (without hemocyte) was first mixed with 40 μL of PBS, and 5 μL of hemolymph solution was used for analysis. Protein concentration of each sample was measured by Nanodrop (A280).

### Glucose, Trehalose, and Adenosine Measurements

The levels of glucose, trehalose, and adenosine were determined in *B*. *mori* hemolymph using colorimetric methods with a glucose assay kit (Cell Biolabs, Inc.), trehalose microplate assay kit (Cohesion Biosciences, Ltd.), and adenosine assay kit (Fluorometric), respectively. The detailed procedures have been described previously (19).

### ATP Analysis

The level of ATP in the samples was assessed using an ATP determination kit (Molecular Probes). Virus-infected BmN cells (2 × 10^5^) were collected by centrifugation at 7,500 × g at 4°C for 1 min. Cells were lysed with 200 μL of cell culture lysis reagent (25 mM Tris-phosphate (pH 7.8), 2 mM DTT, 2 mM 1,2-diaminocyclohexane-N,N,N,N -tetraacetic acid, 10% glycerol, 1% Triton® X-100) and centrifuged at 14,000 rpm for 3 min to remove cell debris. To quantify ATP, 10 μL of collected supernatant or hemolymph was transferred to a 96-well black opaque plate that contained 90 μL of the standard assay solution. Standard solutions of ATP were prepared in the same manner. The reaction was carried out at 28°C, after which the relative light units (RLUs) of the sample and standard solution were simultaneously measured using a SpectraMax Gemini EM Microplate Reader at a maximum emission of 560 nm. A standard curve was plotted using the measured RLUs.

### Statistical Analysis

The Ct values obtained from the RT-qPCR assay were normalized using the 2^−ΔΔCt^ method; the 18 S ribosomal RNA (rRNA) gene was used as the reference gene (27). Comparisons between two groups were performed using Student's *t*-test, with *P* < 0.05 indicating a significant difference (marked with an ^*^ in the figures). Significance between three groups was analyzed by ANOVA with Tukey's HSD *post-hoc* test, and different letters indicate significant differences (*P* < 0.05).

## Results

### Different Responses of Glycolytic Gene Expressions and ATP Synthesis Upon Permissive and Non-permissive Infections

**Figure 1:**
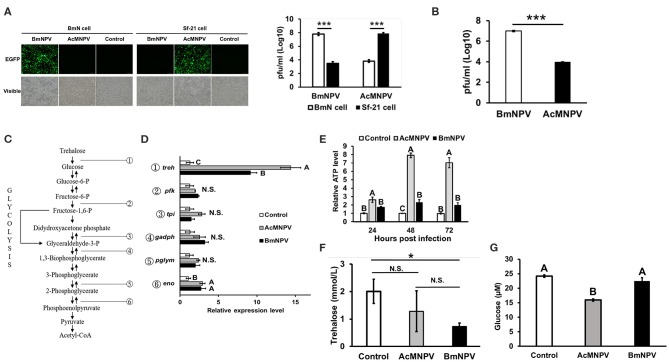
Virus-host tropisms and host glycolytic activities upon AcMNPV and BmNPV infection. **(A)** Virus titers were determined by fluorescence intensity and by qPCR analysis at 48 hpi in BmN and Sf-21 cells infected with AcMNPV or BmNPV. **(B)** Virus titers quantification by qPCR in AcMNPV- or BmNPV infected *B. mori* larvae at 48 hpi **(C)** Summary of glycolytic and citrate cycle enzymes in insects. **(D)** RT-qPCR analysis of glycolytic genes trehalase-2 (*treh*), phosphofructokinase (*pfk*), triose phosphate isomerase (*tpi*), glyceraldehyde 3-phosphate dehydrogenase (*gapdh*), phosphoglyceromutase (*pglym*), and enolase (*eno*) in BmN cells at 48 h after infection with AcMNPV or BmNPV. All of the results were normalized to expression of the 18 S rRNA gene and non-infected control (ΔΔCt). **(E)** ATP levels were measured at 48 hpi in AcMNPV- or BmNPV-infected cells. All of the results were normalized to those in the non-infected control. Hemolymph trehalose **(F)** and glucose **(G)** levels in *B. mori* larvae were measured at 48 hpi; control larvae were injected with 1X PBS. All the values are the mean ± SEM of three **(A,C,G)** or four **(E,F)** replicates. Significances of D, E, F, and G were determined by one-way ANOVA with Tukey's HSD *post-hoc* analysis; different letters for the treatment group indicate significant differences at *P* < 0.05. Student's *t*-test was used for the analysis of A, B, F, *P < 0.05, ***P < 0.001.

It is known that AcMNPV and BmNPV have similar genomic compositions but different host tropisms. To verify their infection capabilities in the present study, Sf-21 and BmN cells were infected with both viruses, and virus titers were calculated after 48 h of infection. The results showed that increased amounts of the viruses were only observed in the BmNPV-infected BmN cells or AcMNPV-infected Sf-21 cells (Figure 1A). Viral titers increased by ~100-fold compared with the non-permissive infection at 48 h after infection. The results also showed that under *in vivo* conditions, increased viral titer were only observed in *Bombyx mori* larvae injected with BmNPV but not in those injected with AcMNPV (Figure 1B).

To assess the response of glycolytic gene expressions under permissive and non-permissive infection, the transcription levels of genes involved in glycolysis were evaluated by real-time quantitative polymerase chain reaction (qPCR) after infecting BmN cells with AcMNPV or BmNPV (Figure 1C). Notably, expression of *treh* was induced by AcMNPV or BmNPV infection, but the induction level in AcMNPV-infected cells was significantly higher than that in BmNPV-infected cells (Figure 1D). No difference between the control and both baculovirus-infected cells was found for other glycolytic genes, including *pfk, tpi, gadph*, and *pglym*; *eno* showed increased transcription after infection, but with no difference between AcMNPV and BmNPV infection. In addition, ATP production after AcMNPV infection significantly increased from 24 to 72 h post infection (Figure 1E). Comparing to AcMNPV infection, BmNPV infection only significantly induced ATP level at 48 h post infection. These results indicated that the expression of a glycolytic gene, *treh*, as well as the production of ATP, was significantly induced by infection with both baculoviruses but was relatively higher in non-permissive AcMNPV-infected cells.

To further confirm these *in vitro* results, we compared circulating trehalose and glucose levels in infected larvae. Although no significant difference by ANOVA was observed for circulating trehalose between PBS-injected larvae and both virus-infected larvae (*P* = 0.07), the level of released trehalose in BmNPV-infected larvae tended to be lower than that in PBS-injected groups (Figure 1F, *P* < 0.05 *t*-test). In addition, the level of glucose was lowest in AcMNPV-infected larvae compared to in BmNPV-infected larvae or the PBS treatment control (Figure 1G). These *in vivo* results demonstrate different glycolytic activities between permissive and non-permissive infection conditions.

### Inhibition of Glycolysis Enhances AcMNPV Replication in a Non-permissive Host

**Figure 2:**
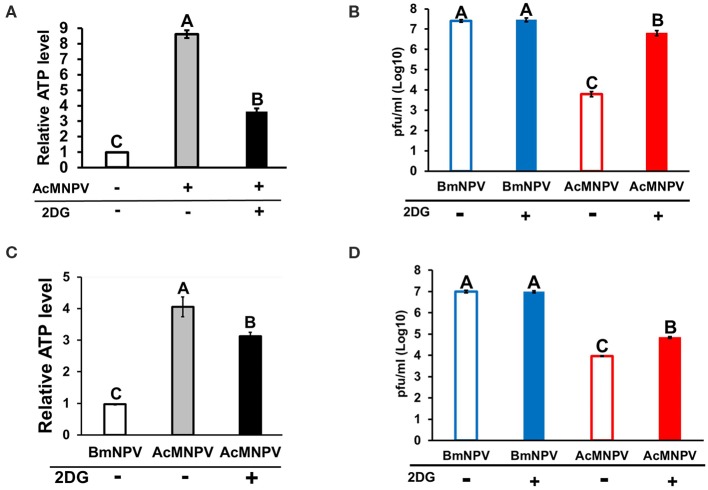
Inhibition of glycolysis by 2-deoxy-D-glucose (2DG) treatment resulted in decreased ATP levels and increased AcMNPV replication in BmN cells and larvae. The ATP level was measured at 48 h postinfection (hpi) after 2DG treatment in BmN cells **(A)** and larvae **(C)**; the values were normalized to those in non-infected control BmN cells and BmNPV-infected larvae, respectively. Virus titers were estimated at 48 hpi in BmN cells **(B)** and larvae **(D)**; BmNPV treatment was used as the positive control. All values are shown as the mean ± SEM of four replicates for ATP measurements and three replicates for virus titer. Significance was determined by one-way ANOVA with Tukey's HSD *post-hoc* analysis; different letters for the treatment group indicate significant differences at *P* < 0.05.

Because BmN cells displayed higher ATP production upon AcMNPV infection, we sought to understand whether such metabolic induction is a host physiological response for enhancing the antiviral immunity against AcMNPV replication or is induced by virus infection for virus replication. To address this issue, we treated AcMNPV-infected BmN cells with the glycolytic inhibitor 2-deoxy-D-glucose (2DG) (28). The results showed that 2DG successfully suppressed ATP production in AcMNPV-infected cells (Figure 2A), and such suppression increased the AcMNPV titer in non-permissive BmN cells, though infection capacity was still lower than in BmNPV infection (Figure 2B). To confirm the *in vitro* results, we conducted the same experiment under *in vivo* conditions. We first confirmed that AcMNPV infection induced higher ATP production than did BmNPV infection (Figure 2C) in larvae, which was shown in BmN cells (Figure 1E), and that ATP production can be further decreased by 2DG treatment. Moreover, glycolysis suppression by 2DG injection resulted in a significant increase in AcMNPV titer in non-permissive *B. mori* larvae (Figure 2D). Notably, 2DG treatments did not influence the BmNPV titers in permissive BmN cells or larvae (Figures 2B,D). Our results show that this glycolytic activation in *B. mori* upon AcMNPV infection indeed plays an important role in preventing AcMNPV replication. Hence, suppression of glycolysis by 2DG treatment increased the AcMNPV replication capacity in its non-permissive host.

### Adenosine Signaling Is Involved in Metabolic Induction Upon Baculovirus Infection

**Figure 3:**
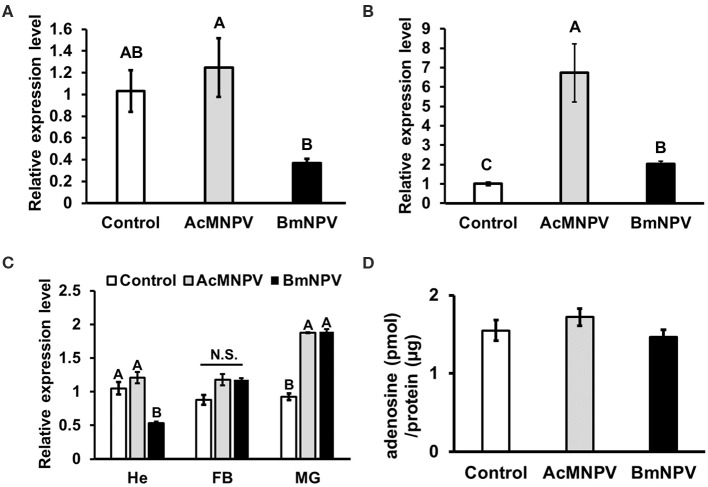
Regulation of adenosine signaling upon AcMNPV and BmNPV infection. RT-qPCR analyses of *AdoR* expression in *B. mori* larvae **(A)** BmN cells **(B)** as well as in different immune organs **(C)** upon AcMNPV or BmNPV infection at 48 h postinfection (hpi). **(D)** Measurement of hemolymph adenosine levels of larvae injected with PBS (control), AcMNPV or BmNPV at 48 hpi. All values are shown as the mean ± SEM of three replicates for qPCR and four replicates for adenosine measurement. Significance was determined by one-way ANOVA with Tukey's HSD *post-hoc* analysis; different letters for the treatment group indicate significant differences at *P* < 0.05. MG, midgut; HE, hemocyte; FB, fat body.

Previous studies in *Drosophila* have demonstrated that adenosine signaling regulates glycolytic activity upon pathogenic infection (19, 21). We examined the temporal and spatial expression profiles of *Bombyx AdoR* by using SilkDB 3.0 database (https://silkdb.bioinfotoolkits.net) (29). We found that *AdoR* is expressed ubiquitously from larval to adult stages, and it is also detectable in immune organs including hemocyte, midgut and fat body. To confirm the involvement of adenosine signaling upon baculovirus infection, we compared the expression level of adenosine receptor (*AdoR*) after AcMNPV and BmNPV infection. *AdoR* expression showed no difference between non-infected larvae and AcMNPV-infected larvae, but both were higher than in BmNPV-infected larvae (Figure 3A). In BmN cells, *AdoR* expression increased after AcMNPV or BmNPV infection but was highest in AcMNPV-infected cells (Figure 3B). In addition, we examined the *AdoR* expression profiles in different immune organs (hemocyte, fat body, midgut), and results showed that BmNPV infection significantly suppressed AdoR expression in hemocyte (Figure 3C). Both virus infection significantly induced AdoR expressions in midgut but no impact on the AdoR expression in the fat body. The results revealed different profiles of *AdoR* transcription under *in vivo* and *in vitro* conditions, which might be due to different tissue-specific responses and the difference in complexity between whole larvae and BmN cells. Such transcriptional tissue-specific responses were also observed upon BmCPV infections in *Bombyx mori* (30). The lower *AdoR* expression in the hemocyte of BmNPV-infected larvae suggested that BmNPV suppresses the *AdoR* expression for compromising the host immune defense. Since *AdoR* in insect hemocyte has known playing the important roles on energy metabolism and cellular immune responses upon the bacterial and virus infections (21, 31) as well as hematopoiesis (32). Notably, the same patterns of *AdoR* expression in BmNPV-infected larvae and BmN cells always being lower than in AcMNPV infection were found. We further measured the extracellular adenosine level in the hemolymph of infected larvae but found no significant difference between the PBS injection control and AcMNPV- and BmNPV-infected larvae (Figure 3D). Our results indicate that adenosine signaling is lower under BmNPV infection than under AcMNPV infection.

**Figure 4:**
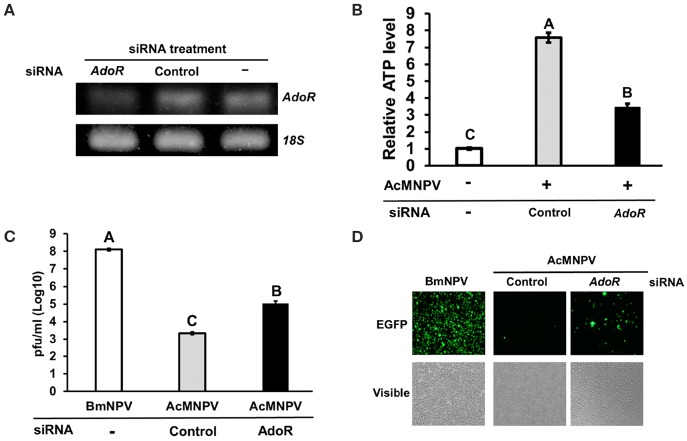
*AdoR* RNAi suppressed ATP induction and increased AcMNPV titers in BmN cells. **(A)** Knockdown efficiency of *AdoR* siRNA treatment in BmN cells by RT-PCR analysis. **(B)** The ATP level was measured at 48 h postinfection (hpi) in *AdoR* and control siRNA-treated cells; values were normalized to those in the non-infected control. Virus titers were determined by qPCR **(C)** and fluorescence intensity **(D)** at 48 hpi in *AdoR-* and control siRNA-treated cells. All values are the mean ± SEM of four replicates for ATP level and three replicates for virus titer measurements. Significance was determined by one-way ANOVA with Tukey's HSD *post-hoc* analysis; different letters for the treatment group indicate significant differences at *P* < 0.05.

To understand whether adenosine signaling regulates host metabolism and influences the capacity of AcMNPV replication in its non-permissive host, we inhibited *AdoR* expression by RNAi in AcMNPV-infected BmN cells and measured the ATP level and AcMNPV titer. *AdoR* transcription was successfully silenced after 48 h of transfection with *AdoR* siRNA (Figure 4A); moreover, induction of ATP levels upon AcMNPV infection was significantly decreased in BmN cells (Figure 4B). Notably, this metabolic suppression by *AdoR* RNAi significantly increased the AcMNPV titer compared with control siRNA treatment cells, but the titer was still lower than that in BmNPV infection (Figure 4C). The result was also visible by observing GFP (expressed from the viral sequence) signals under a fluorescence microscope. The GFP signal in *AdoR* siRNA-treated cells was greater than that in control-treated cells (Figure 4D).

**Figure 5:**
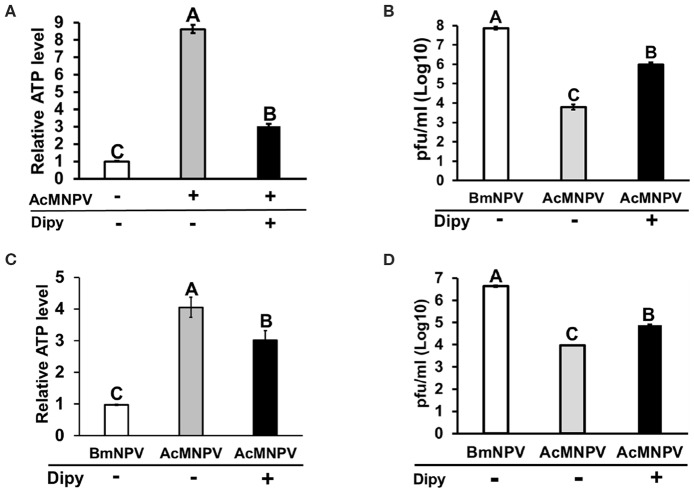
Inhibition of adenosine transport by dipyridamole (Dipy) treatment decreased the ATP level, resulting in an increase in AcMNPV replication in BmN cells and larvae. The ATP level was measured at 48 h postinfection (hpi) with Dipy treatment in BmN cells **(A)** and larvae **(C)**; values were normalized to those in the non-infected control BmN cells and BmNPV-infected larvae, respectively. Virus titers were estimated at 48 hpi in BmN cells **(B)** and larvae **(D)**; BmNPV treatment represented the positive control. All values are shown as the mean ± SEM of four replicates for ATP level and three replicates for virus titer measurements. Significance was determined by one-way ANOVA with Tukey's HSD *post-hoc* analysis; different letters for the treatment group indicate significant differences at *P* < 0.05.

Inhibiting efflux transport of adenosine under stress conditions has been reported to decrease *AdoR* signaling (19, 33). To further confirm the RNAi results, we pharmaceutically blocked adenosine transport by treating cells with the equilibrative nucleoside transporter (ENT) inhibitor dipyridamole (Dipy). Blocking adenosine transportation suppressed ATP induction upon AcMNPV infection (Figure 5A) and significantly increased the AcMNPV titer in BmN cells (Figure 5B). We conducted the same experiment under *in vivo* conditions by injecting infected larvae with Dipy and observed the same results, whereby Dipy decreased ATP induction upon AcMNPV infection, increasing AcMNPV infective capacity in *B. mori* larvae (Figures 5C,D). We conclude that *AdoR* indeed regulates host metabolic induction upon AcMNPV infection and is essential for the host antivirus response.

### Metabolic Activation Is Essential for the Antivirus Immune Response

**Figure 6:**
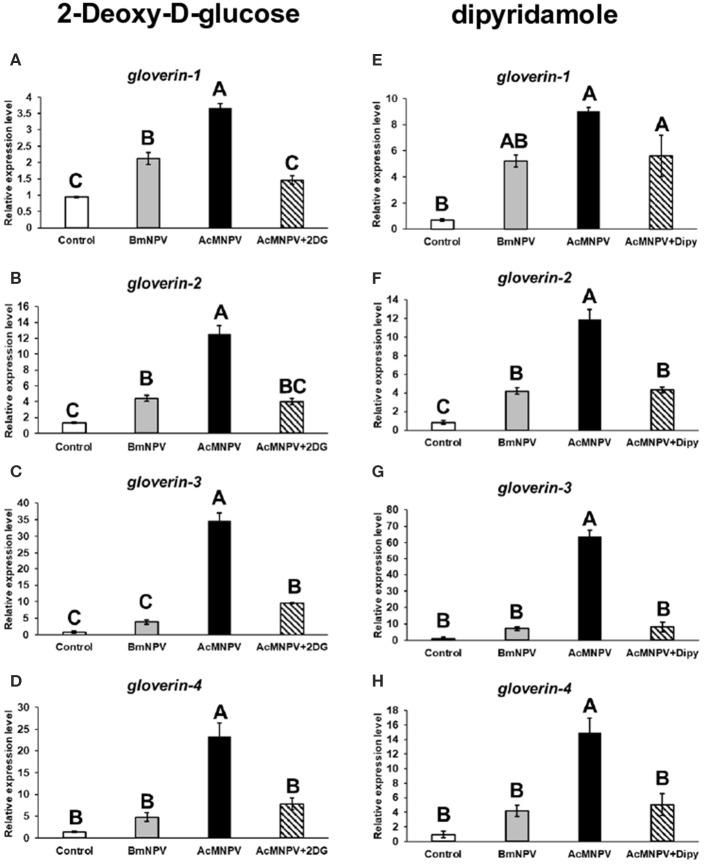
Antivirus protein expression was regulated by *AdoR*-meditated metabolic activation upon AcMNPV infection. The expression levels of four gloverin genes were analyzed by RT-qPCR at 48 h postinfection (hpi) in larvae infected with BmNPV or AcMNPV and cotreated with 2DG **(A–D)** or Dipy **(E–H)**. Control larvae were injected with 1X PBS. All values are the mean ± SEM of three replicates. Significance was determined by one-way ANOVA with Tukey's HSD *post-hoc* analysis; different letters for the treatment group indicate significant differences at *P* < 0.05.

Antimicrobial peptides (AMPs) have been reported to be involved in antivirus immune reactions in insects (13, 34). Of these, gloverin was shown that highly induced in the BmNPV-resistant strain of *B. mori* upon infection and suppressed by AcMNPV infection in *Spodoptera exigua* larvae (35, 36). Preincubation of Sf-9 cells with gloverin peptides also reduces the production of budded AcMNPV virus (37). In addition, suppression of gloverin expression by RNAi increased the AcMNPV replication in BmN cells (data not shown). To confirm that metabolic induction is an important factor enhancing the antivirus response to restrict AcMNPV permissiveness in *B. mori*, we inhibited glycolysis by injecting 2DG into infected larvae and assessed transcription of four *gloverin* genes (Figures 6A–D). The expression levels of the four *gloverin* genes were increased after infection by both baculoviruses but relatively higher with AcMNPV. Notably, 2DG treatment significantly decreased induction of all *gloverin* transcripts, confirming our hypothesis that metabolic activation upon AcMNPV infection is essential for supporting the immune response against infection. Furthermore, to again prove that adenosine signaling regulates host metabolic activation to support the antivirus response, we injected Dipy to block adenosine transport in infected larvae and measured expression of the four *gloverin* genes (Figures 6E–H). Except for *gloverin*-1, the other three *gloverins* showed similar results: Dipy injection significantly suppressed expression due to AcMNPV infection. Our results demonstrate that metabolic induction regulated by adenosine signaling is critical for the antiviral immune response in *B. mori*.

### Adenosine Signaling Is a Conservative Mechanism Modulating the Permissiveness of Baculovirus Infection in *Spodoptera frugiperda* Cells

**Figure 7:**
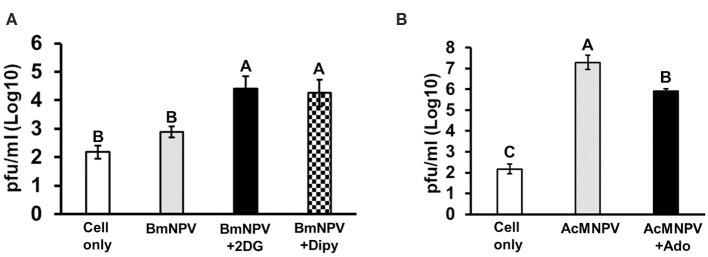
Glycolysis and adenosine signaling in Sf-21 cells regulate the permissiveness of baculovirus infection. Sf-21 cells were preincubated with 2DG and Dipy **(A)** and adenosine (Ado) **(B)** before BmNPV or AcMNPV infection, respectively. The virus titers were estimated at 48 h postinfection. “Cell only” indicates cells without virus and drug treatments. All values are shown as the mean ± SEM of three replicates. Significance was determined by one-way ANOVA with Tukey's HSD *post-hoc* analysis; different letters for the treatment groups indicate significant differences at *P* < 0.05.

To demonstrate that our observations are not restricted to *B. mori*, we tested the role of adenosine signaling in another lepidopteran model, *S. frugiperda* Sf-21 cells. We obtained the same results, showing that inhibition of glycolysis and adenosine transport in Sf-21 cells increased the BmNPV replication in its non-permissive host (Figure 7A). Alternatively, enhancement of adenosine signaling in Sf-21 cells by applying adenosine led to a significant decrease in the AcMNPV infection capacity in its permissive host (Figure 7B). Our results indicated that inhibition of adenosine signaling resulted in a decreased glycolytic activity and antivirus reaction, which increased the baculovirus infective capacity in its non-permissive host; conversely, induction of adenosine signaling enhanced the host antivirus reaction, which decreased the AcMNPV propagation in Sf-21 cells.

## Discussion

AcMNPV has broader host range in comparing to BmNPV, which has only one permissive host, silkworm and its derived cell line Bm cells. Interestingly, despite having a wide range of host, AcMNPV is not able to achieve successful infection in Bm cells, making Bm cells non-permissive to AcMNPV (5, 7). Host tropism may be determined by the following factors: the ability of baculoviruses to enter host cells, to achieve normal viral gene expression during infection, and to utilize host cellular machinery to complete the infection procedure (6, 38). Many viral genes involved in host range determination have also been identified, including p143, p35, and hcf-1. Substitution of 2 amino acids in AcMNPV *p143* enabled AcMNPV replication in Bm5 cells. Blocking cell apoptosis and activating origin-specific DNA replication in AcMNPV can also alter host specificity, demonstrating that virus-host interactions can also alter host ranges in some baculoviruses. Baculoviruses produce two distinct types of virions during the infection cycle: budded viruses (BV), which are responsible for systematic infection within hosts, and occlusion derived viruses (ODV), which are responsible for spreading infection to other susceptible species (39). Infective efficiency might be variable by oral delivery of ODV, since several antiviral proteins such as Bmlipase-1 and BmSP-2 are highly expressed in midgut, and virus also needs to passes through the host's peritrophic membrane for causing the systemic infection (39–41). Taking into consideration, our study mainly injected BV into the hemocoel of silkworms.

Previous studies on the regulation of host tropism of AcMNPV and BmNPV have mostly focused on how viruses modulate the cellular function of the host to establish successful propagation. Conversely, relatively few studies have investigated the host physiological response, which is important for anti-baculovirus reactions. Our results showed that expression of the glycolytic genes *treh* and *eno* as well as ATP production increased after BmNPV infected BmN cells (Figure 1). These results are consistent with previous observations on BmNPV-infected BmN cells or AcMNPV-infected Sf-9 cells, which showed increased citric acid expression, TCA cycle activity, and ATP levels after infection (11, 12). It was later concluded that virus induces metabolic activity of a permissive host due to the requirement of a large energy supply for baculovirus replication (9, 13). Regardless, the fact that the immune system of the host also requires a higher energy supply for antivirus immune responses should not be overlooked. In fact, our results showed that metabolic induction was more dramatic under non-permissive infection conditions. Both the *treh* expression level and ATP production in AcMNPV-infected BmN cells and larvae were significantly higher than under BmNPV infection (Figures 1, 2). It was reported previously that AcMNPV infection of permissive host (Sf-9 cells) caused cell enlargements and resulted in increasing intracellular ATP level by 50–80% (42). In the present study, intracellular ATP level increased by 6 to 7-fold (600–700%) in AcMNPV infection of the non-permissive host (BmN cells). This increased ATP level was far more than that in the permissive cell line, suggesting that it was not likely contributed by cell enlargement after infection.

Trehalose circulation and consumption of glucose were also higher in AcMNPV-infected larvae (Figure 1). Moreover, instead of promoting virus infection, as in previous studies describing permissive infection, this metabolic induction under non-permissive infection appears to be a host physiological response against virus replication. Increased AcMNPV titers and suppressed *gloverin* induction were observed after applying the glycolytic inhibitor 2DG to infected BmN cells and larvae (Figures 2, 6). In general, increased energy consumption upon pathogenic challenge is essential for supporting both cellular and humoral immune responses (18). In particular, the production of AMPs is usually dramatically and rapidly enhanced during infection, and it has been shown that activation of the IMD and Toll pathways as well as *drosomycin* overexpression have significant metabolic impacts in *Drosophila*, such as reduced glycogen and triglyceride stores (43–45). Our results show that activation and reallocation of energy supply toward the immune system is necessary for anti-baculovirus reactions.

Our results demonstrated that adenosine signaling is a key molecular mechanism regulating metabolic induction upon virus infection. Suppressing *AdoR* expression in BmN cells or inhibiting adenosine transport in BmN cells and larvae by RNAi affected ATP production and *gloverin* expression, resulting in increased AcMNPV-infected capability in non-permissive hosts (Figures 4–6). As a signaling molecule, adenosine is known to be involved in various stress responses, including immune reactions. Extracellular adenosine can be derived from the degradation of extracellular ATP or ADP release by damaged cells, or it can be converted from intracellular ATP and exported to the extracellular space via ENTs (46). Increased ATP synthesis under infection leads to higher extracellular adenosine levels, activating AdoR signaling, which regulates several immune responses, such as inflammatory cytokine production in mammals and hematopoiesis and phagocytosis in *Drosophila* (21, 47, 48). Additionally, our results are consistent with previous findings demonstrating that adenosine signaling regulates carbohydrate metabolism and energy distribution during bacterial and wasp infection in *Drosophila* (18, 19). Based on previous data and those from our present study, which used two different lepidopteran models, we conclude that adenosine signaling may be a conserved mechanism that modulates host metabolism and immune reactions during pathogenic infection.

Notably, we discovered that *AdoR* expression (Figure 3), *treh* expression (Figure 1D), and ATP levels (Figure 1E) in BmNPV-infected cells or larvae were significantly lower than in AcMNPV infection. As our previous study demonstrated that BmNPV infection resulted in strong miRNA production in *B. mori* (1), we speculated that BmNPV infection may block host adenosine signaling by stimulating miRNA against *AdoR* expression, further suppressing metabolic activation and the antivirus response. We reexamined the transcriptome data from our previous study and found several potential miRNAs targeting *AdoR* (Table S2). Because induction of host miRNA expression by viral challenge may be a host antiviral response or it could be triggered by the virus for host physiology remodeling, further study will be needed to characterize the major miRNA involved in regulating *AdoR* signaling upon BmNPV infection.

Our experimental results confirm that adenosine signaling affects glycolytic and energy synthesis in *B. mori*, affecting the host antivirus immune response and restricting the host specificity of AcMNPV. Thus, our study provides a basis for future investigations on the association between host physiological responses and baculovirus infection, and the findings may also be relevant for pest control management.

## Data Availability Statement

The datasets analyzed in this article are not publicly available. Requests to access the datasets should be directed to runwu@ntu.edu.tw.

## Author Contributions

Y-HL, C-CT, VB, C-KT, PC, and Y-LW: guarantors of integrity of entire study, study concepts, and manuscript preparation. Y-HL, C-CT, C-KT, PC, C-HL, and Y-LW: study design, data acquisition/analysis, literature research, and manuscript preparation. Y-HL, C-CT, VB, CW, and Y-LW: data acquisition/analysis, manuscript editing, and revision. All authors reviewed the manuscript.

## Conflict of Interest

The authors declare that the research was conducted in the absence of any commercial or financial relationships that could be construed as a potential conflict of interest.

## Supplementary Material

Table S1 List of qPCR primers.

Table S2 List of predicted *AdoR* miRNAs in BmNPV-infected BmN cells.

Figure S1 Cytotoxicity assessment of Dipy, 2DG and adenosine treatments. The BmN **(A)** and Sf-21 cells **(B)** were treated with DMSO (0.05%, labeled as control or ctr), 2-Deoxy-D-glucose (10 mM, labeled as 2DG), Dipyridamole (20 μM, labeled as Dipy) and adenosine (10 mM, labeled as Ado) for 24, 48, and 72 h, and cells were stained with propidium iodide (50 μg/mL) for labeling the dead cells. The quantification of live and dead cells was conducted by flow cytometry in the PE-A (585 ± 40 nM) channel using ACEA NovoCyteTM 3000, and 10,000 events were quantified for comparison. All values of bar graph are shown as the mean ± SEM of three replicates. Kruskal-Wallis test was used for statistical analysis, and results suggested that no significant difference of live or dead cell numbers among all the treatment in both cell line.
