# Supplementary material for: Adenosine Receptor Modulates Permissiveness of Baculovirus (Budded Virus) Infection via Regulation of Energy Metabolism in Bombyx mori
Source: Front Immunol. 2020 Apr 28;11:763. doi: 10.3389/fimmu.2020.00763 (PMC7198810; doi:10.3389/fimmu.2020.00763)
Supplement: Table S1 — List of qPCR primers. [file Data_Sheet_1.PDF]

Supplementary Materials and Methods

Table S1. The list of qPCR primers

Primer Name	Primers (5'→3')
Treh-F	5'-TACTGGTGGGATTGACGACG-3'
Treh-R	5'-ATTCGTGCGGTCCAATAGCT-3'
Pfk-F	5'-GTCTTCATTGGCTCGTTGAG-3'
Pfk-R	5'-AGGATACGCCGCAATGGACG-3'
Tpi-F	5'-ACCCGCTATGATCAAAGACA-3'
Tpi-R	5'-CAACTTCCTCAGTCTTGCCA-3'
Gadph-F	5'-AGTATGATTCCACCCATGGC-3'
Gadph-R	5'-TCCCTCCAAGTGAGCAGATG-3'
Pglym-F	5'-CTTCTGTGGATGGTTCGACG-3'
Pglym-R	5'-TGCCTCTCGTTCAATCTCCA-3'
Eno-F	5'-ACAAACCCTAAGCGTATCG-3'
Eno-R	5'-AACTACCAGGTCGGCAATA-3'
adoR-F	5'-TCTGGTTGCTTTAGTGGCAG-3'
adoR-R	5'-ACTGTGAAGAGACACGCATG-3'
Gloverin-1F	5'-CACGACTTTGTCACTTGG-3'
Gloverin-1R	5'-GCTTACGAGGCAAGAATG-3'
Gloverin-2F	5'-GTCTTGAGGAGCGAAACT-3'
Gloverin-2R	5'-GTCATAACAAAGCACGAG-3'
Gloverin-3F	5'-ACTAGCCAAACCACAAAC-3'
Gloverin-3R	5'-GATGGGATTGTGTTGACA-3'
Gloverin-4F	5'-ATTGGGAGGACGAAGAAG-3'
Gloverin-4R	5'-TGCAGAGTGAAAGTATCG-3'
