# Supplementary material for: Adenosine Receptor Modulates Permissiveness of Baculovirus (Budded Virus) Infection via Regulation of Energy Metabolism in Bombyx mori
Source: Front Immunol. 2020 Apr 28;11:763. doi: 10.3389/fimmu.2020.00763 (PMC7198810; doi:10.3389/fimmu.2020.00763)
Supplement: Table S2 — List of predicted AdoR miRNAs in BmNPV-infected BmN cells. [file Data_Sheet_2.PDF]

Supplementary Materials and Methods

Table S2. The list of AdoR miRNA prediction from BmNPV-infected BmN cell

miRNA	Accession No.	Predicted binding sites	Score
bmo-miR-6498-5p	MIMAT0025323	3'UTR	145
bmo-miR-317-3p	MIMAT0007906	3'UTR	140
bmo-miR-2753	MIMAT0012604	3'UTR	142
bmo-miR-3369	MIMAT0015555	3'UTR	145
