# Supplementary figures and images for: Adenosine Receptor Modulates Permissiveness of Baculovirus (Budded Virus) Infection via Regulation of Energy Metabolism in Bombyx mori
Source: Front Immunol. 2020 Apr 28;11:763. doi: 10.3389/fimmu.2020.00763 (PMC7198810; doi:10.3389/fimmu.2020.00763)

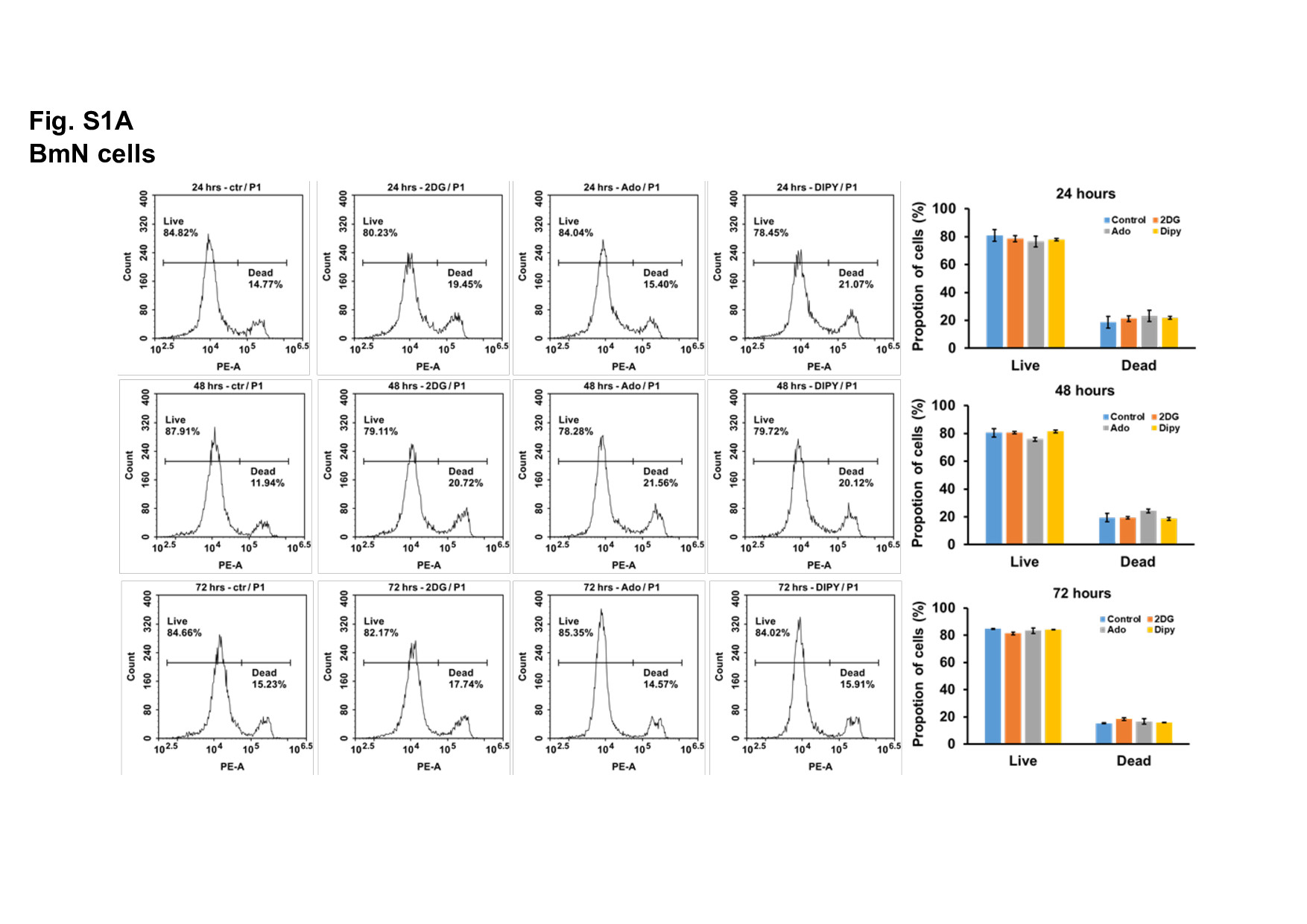

Supplement: Supplementary file 3 [file Image_1.JPEG]

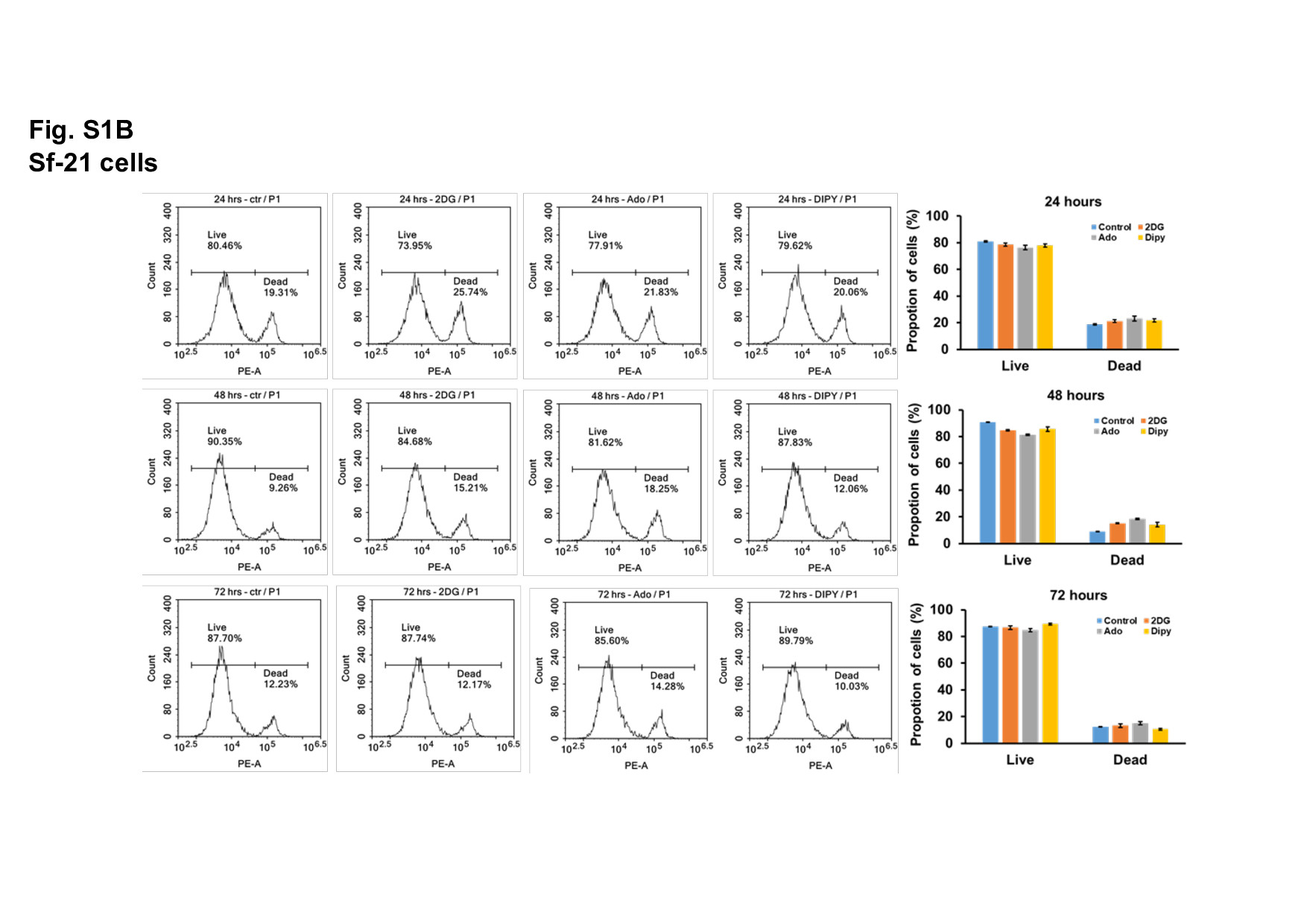

Supplement: Figure S1 — Cytotoxicity assessment of Dipy, 2DG and adenosine treatments. The BmN (A) and Sf-21 cells (B) were treated with DMSO (0.05%, labeled as control or ctr), 2-Deoxy-D-glucose (10 mM, labeled as 2DG), Dipyridamole (20 μM, labeled as Dipy) and adenosine (10 mM, labeled as Ado) for 24, 48, and 72 h, and cells were stained with propidium iodide (50 μg/mL) for labeling the dead cells. The quantification of live and dead cells was conducted by flow cytometry in the PE-A (585 ± 40 nM) channel using ACEA NovoCyteTM 3000, and 10,000 events were quantified for comparison. All values of bar graph are shown as the mean ± SEM of three replicates. Kruskal-Wallis test was used for statistical analysis, and results suggested that no significant difference of live or dead cell numbers among all the treatment in both cell line. [file Image_2.JPEG]
