# Supplementary material for: Adenosine Receptor Modulates Permissiveness of Baculovirus (Budded Virus) Infection via Regulation of Energy Metabolism in Bombyx mori
Source: Front Immunol. 2020 Apr 28;11:763. doi: 10.3389/fimmu.2020.00763 (PMC7198810; doi:10.3389/fimmu.2020.00763)
Supplement: Supplementary file 5 [file Data_Sheet_3.PDF]

Supplementary Materials and Methods

miRNA target prediction

miRNAs induced by BmNPV infection in BmN cells were identified in our previous article (Chen et al., 2018). To identify miRNAs that target AdoR, an open-source software based on the miRanda algorithm was employed to predict miRNA targets (John et al., 2004). The miRNAs with high match scores chosen for analysis are listed in Table S2.

References

Chen, Y.W., Wu, C.P., Wu, T.C., Wu, Y.L., 2018. Analyses of the transcriptome of Bombyx mori cells infected with either BmNPV or AcMNPV. Journal of Asia-Pacific Entomology 21, 37-45.

John, B., Enright, A.J., Aravin, A., Tuschl, T., Sander, C., Marks, D.S., 2004. Human MicroRNA targets. PLoS Biol 2, e363.
